# Preoperative uric acid predicts in-hospital death in patients with acute type a aortic dissection

**DOI:** 10.1186/s13019-020-1066-9

**Published:** 2020-01-15

**Authors:** Yiran Zhang, Xingjie Xu, Yuan Lu, Lei Guo, Liang Ma

**Affiliations:** Department of Cardiovascular Surgery, First Affiliated Hospital, College of Medicine, Zhejiang University, Hangzhou, 310003 China

**Keywords:** Uric acid, Acute type A aortic dissection, In-hospital mortality

## Abstract

**Background:**

The present study aimed to evaluate the value of admission serum uric acid (UA) level in predicting in-hospital risk of death in patients with acute type A aortic dissection (AAAD).

**Methods:**

From January 2016 to June 2019, 186 consecutive patients with AAAD who underwent thoracic aortic surgery were retrospectively studied. Serum UA levels were measured on admission. Forward conditional logistic regression was performed to identify independent risk factors for in-hospital death. Receiver operating characteristic (ROC) analysis was performed to assess the most clinical useful level of serum UA for predicting postoperative in-hospital mortality.

**Results:**

Increased level of serum UA was found in non-survivors compared with those survived (446 ± 123 vs 371 ± 111 umol/L, *p* < 0.001). Age (OR = 1.063, 95% CI 1.016–1.112, *p* = 0.009), UA (OR = 1.006, 95% CI 1.002–1.010, *p* = 0.002), D-dimer (OR = 1.025, 95% CI 1.005–1.013, *p* = 0.012), operation time (OR = 1.009, 95% CI 1.005–1.013, *p* < 0.001) and extent of aortic replacement (OR = 0.412, 95% CI 0.220–0.768, *p* = 0.005) were identified as independent risk factors of in-hospital mortality in AAAD patients. The best cut-off value of admission serum UA in predicting in-hospital mortality was determined to be 415 umol/L. Subgroup analysis showed that in the subgroup of total arch replacement, UA was significantly associated with in-hospital death (OR = 1.010, 95% CI 1.005–1.015, *p* < 0.001), while in patients underwent ascending aorta replacement or hemiarch replacement, the relationship was no longer significant (OR = 1.001, 95% CI 0.996–1.006, *p* = 0.611).

**Conclusions:**

Elevated serum UA level on admission is an independent predictor of in-hospital mortality in patients with AAAD.

## Introduction

Despite improvements in surgical technique, the early mortality after surgery for acute type A aortic dissection (AAAD) remained as high as high as 8–25% [1, 2]. Several laboratory indexes on admission such as D-dimer [3–5], C reactive protein (CRP) [3], fibrinogen [6], platelet count [7], and white blood cell count (WBC) [8] have been proven to be associated with early mortality in patients with aortic dissection.

Uric acid (UA) is the final product of purines metabolism, which is converted from xanthine in an oxidation reaction catalyzed by xanthine oxidoreductase [9]. Several experimental and human studies have shown the association between serum UA level and endothelial dysfunction [10], oxidative stress [11] and systemic inflammation [12]. Epidemiological studies suggested that increased serum UA level was associated with increased cardiovascular disease mortality [13], poorer survival after coronary artery bypass grafting and cardiac valve surgery [14, 15].

Serum UA level has been found associated with total antioxidant capacity and ascending aorta dilatation [16]. It has also been shown that serum UA levels are significantly higher in patients with aortic dissection than those in healthy controls [17]. Those studies suggested that elevated serum UA level might contribute to the pathogenesis of aortic aneurysm and aortic dissection. However, so far, no study has examined the prognostic significance of UA in AAAD, and the relationship between preoperative serum UA and mortality after surgery for AAAD remains unknown.

The present study aimed to evaluate the value of serum UA level in predicting in-hospital risk of death in patients with AAAD.

## Materials and methods

### Study population

This study was approved by the ethics committee of our hospital on 7th July, 2019 (reference number: 2019–1094) and the need for individual consent was waived due to its retrospective nature. We consecutively enrolled 186 patients with AAAD who underwent thoracic aortic surgery between January 2016 and June 2019 at the First Affiliated Hospital, School of Medicine, Zhejiang University in China. Aortic dissection was diagnosed according to medical history, physical examination and computed tomography. The aortic dissection is considered as AAAD if the time from the onset of the symptom to operation is within 14 days.

### Clinical data collection

Clinical variables of enrolled patients was obtained through review of medical records, which included age, gender, medical history (hypertension, diabetes, chronic kidney disease, prior aortic stent implant, prior cardiac surgery, and smoking), the time from onset of the symptoms to operation, vital signs on admission (systolic blood pressure, diastolic blood pressure, and heart rate), ascending aorta diameter (measured by echocardiography preoperatively), number of entry tears (according to the operation note), laboratory data on admission (uric acid, creatine, urea nitrogen, D-dimer, fibrinogen, platelet count, and white blood cell count), organ ischemia (brain, coronary artery involvement, lower limb and eGFR< 60 ml/min/1.73 m^2^). Operative variables included the specific surgical procedure performed, the duration of operation, cardiopulmonary bypass (CPB), aortic cross-clamping (ACC), and hypothermic circulatory arrest (HCA).

The study endpoint was defined as all-cause mortality during hospitalization.

### Surgical procedure

The procedures were performed via median sternotomy. Cardiopulmonary bypass was established with right axillary artery for arterial cannulation site, and right atrium for venous cannulation site. The extent of distal aortic resection was determined based on the location of the entry tear, aortic diameter and the false lumen status of the downstream aorta. Deep hypothermic circulatory arrest (nasopharyngeal temperature 20 °C) with antegrade cerebral perfusion was used in total arch replacement. Bentall procedure or valve-sparing reimplantation was performed in patients with aortic root dilation or an intimal tear at the aortic root level.

### Statistical analysis

Categorical variables are expressed as the number of patients (%). Normally distributed continuous data are presented as mean ± SD, whereas non-normally distributed continuous data are presented as median (interquartile range). Fisher’s exact test or the chi-square test was used to compare categorical variables. The unpaired Student t test was used to compare normally distributed continuous variables, and the Kruskal-Wallis rank test was used to compare non-normally distributed continuous variables.

A forward conditional logistic regression was performed to identify independent risk factors for in-hospital death. Variables were allowed to enter at *p* value< 0.05 and be retained only if they remained significant at *p* value < 0.10.

Receiver operating characteristic (ROC) analysis was performed to assess the most clinical useful level of serum uric acid for predicting postoperative in-hospital mortality. A cute-off value for the highest Youden index (sensitivity+ specificity-1) was identified. Calculation of the area under the receiver operating characteristic curve (AUC) was used to compare the predictive value of different variables. A combined ROC curve was calculated based on the logistic regression.

All statistical analyses were processed using SPSS 19.0 software (SPSS Inc., Chicago, Illinois), and *p* value of < 0.05 was considered statistically significant.

## Results

### Patients’ characteristics and operation data

The present study included 186 consecutive patients with AAAD. Among the 186 patients, 40 patients (21.5%) died after surgery during hospitalization. Clinical features of survivors and non-survivors are summarized in Table 1. Compared with the survivors, the non-survivors were older (55 ± 13 years vs 49 ± 12 years, *p* = 0.016). The onset to operation time (20(14–32) vs 28(19–42) hours, *p* = 0.011) was significantly shorter in death than in survival group. The rate of preoperative renal dysfunction (defined as eGFR< 60 ml/min/1.73 m^2^) was significantly higher in death than in survival group (45% vs 21%, *p* = 0.003). Serum UA (446 ± 123 vs 371 ± 111 umol/L, *p* < 0.001), creatinine (111(77–142) vs 84(68–108) umol/L, *p* = 0.004), blood urea nitrogen (7.19(6.31–9.26) vs 6.66(5.27–8.00) mmol/L, *p* = 0.048), D-dimer (17.9(7.8–38.2) vs 7.6(3.5–16.0) ug/mL FEU, *p* < 0.001) concentrations were significantly higher in death than in survival group. Fibrinogen (1.76(1.16–2.54) vs 2.36(1.67–3.10) g/L, *p* = 0.002) and platelet count (153(115–176) vs 171(139–202) *10^9^/L, *p* = 0.002) were significantly lower in the non-survival group. The non-survival group had a higher rate of total arch replacement (75% vs 52%, *p* = 0.009), and a lower rate of ascending aortic replacement (17% vs 38%, *p* = 0.016). The operation time (540(455–649) vs 406(343–475) min, *p* < 0.001), CPB time (238(198–333) vs 195(162–227) min, *p* < 0.001), ACC time (166(138–208) vs 141(119–179) min, *p* = 0.012) and HCA time (36(29–46) vs 30(0–40) min, *p* < 0.001) were all longer in the non-survival group.

**Table 1:** Clinical characteristics of patients with AAAD

Variable	All patients	Survivor	Non-survivor	*p* value
	(*n* = 186)	(*n* = 146)	(*n* = 40)	
Demographics				
Age (years)	50 ± 12	49 ± 12	55 ± 13	**0.016**
Male, n (%)	149 (80%)	118 (81%)	31 (78%)	0.641
Medical histories, n (%)				
Hypertension	105 (56%)	80 (55%)	25 (63%)	0.384
Diabetes	6 (3%)	5 (3%)	1 (3%)	1
CKD	7 (4%)	7 (5%)	0 (0%)	0.349
Prior aortic stent implant	9 (5%)	8 (5%)	1 (3%)	0.687
Prior cardiac surgery	3 (2%)	3 (2%)	0 (0%)	1
Smoker	72 (39%)	61 (42%)	11 (28%)	0.1
Clinical presentation				
Onset to operation time (hours)	26 (17–39)	28 (19–42)	20 (14–32)	**0.011**
Systolic blood pressure (mmHg)	129 ± 30	129 ± 29	127 ± 32	0.7
Diastolic blood pressure (mmHg)	72 ± 19	72 ± 19	72 ± 22	0.949
Heart rate (beats/min)	80 (68–91)	80 (69–91)	79 (66–88)	0.763
Ascending aorta diameter (mm)	42 (40–50)	42 (39–50)	44 (41–48)	0.231
Entry tears≥2, n (%)	14 (8%)	12 (8%)	2 (5%)	0.738
Laboratory tests on admission				
UA (umol/L)	386 ± 118	371 ± 111	446 ± 123	**< 0.001**
Creatinine (umol/L)	85 (69–117)	84 (68–108)	111 (77–142)	**0.004**
BUN (mmol/L)	6.80 (5.40–8.16)	6.66 (5.27–8.00)	7.19 (6.31–9.26)	**0.048**
D-dimer (ug/mL FEU)	8.7 (3.8–18.2)	7.6 (3.5–16.0)	17.9 (7.8–38.2)	**< 0.001**
Fibrinogen (g/L)	2.18 (1.51–3.03)	2.36 (1.67–3.10)	1.76 (1.16–2.54)	**0.002**
Platelet (10^9^/L)	166 (134–198)	171 (139–202)	153 (115–176)	**0.005**
WBC (10^9^/L)	13.4 (10.7–16.0)	13.2 (10.6–15.7)	14.0 (10.9–16.9)	0.134
Organ ischemia, n (%)				
Brain	10 (5%)	7 (5%)	3 (8%)	0.450
Coronary artery involvement	37 (20%)	27 (18%)	10 (25%)	0.361
Lower limb	45 (24%)	31 (21%)	14 (35%)	0.072
eGFR< 60 ml/min/1.73 m2	49 (26%)	31 (21%)	18 (45%)	**0.003**
Operation profiles				
Extent of aortic replacement, n (%)				
Total arch replacement	106 (57%)	76 (52%)	30 (75%)	**0.009**
Hemiarch replacement	18 (10%)	15 (10%)	3 (8%)	0.823
Ascending aortic replacement	62 (33%)	55 (38%)	7 (17%)	**0.016**
Bentall, n (%)	63 (34%)	50 (34%)	13 (33%)	0.836
Operation time (min)	424 (354–520)	406 (343–475)	540 (455–649)	**< 0.001**
CPB time (min)	200 (167–242)	195 (162–227)	238 (198–333)	**< 0.001**
ACC time (min)	146 (121–187)	141 (119–179)	166 (138–208)	**0.012**
HCA time (min)	31 (0–41)	30 (0–40)	36 (29–46)	**0.001**

Data are represented as median (interquartile range), n(%), or mean ± SD

The bold values indicate statistical significance

*CKD* chronic kidney disease, *eGFR* estimated glomerular filtration rate, *UA* uric acid, *BUN* blood urea nitrogen, *WBC* white blood cell count, *CPB* cardiopulmonary bypass, *ACC* aortic cross-clamping, *HCA* hypothermic circulatory arrest

**Fig. 1:**
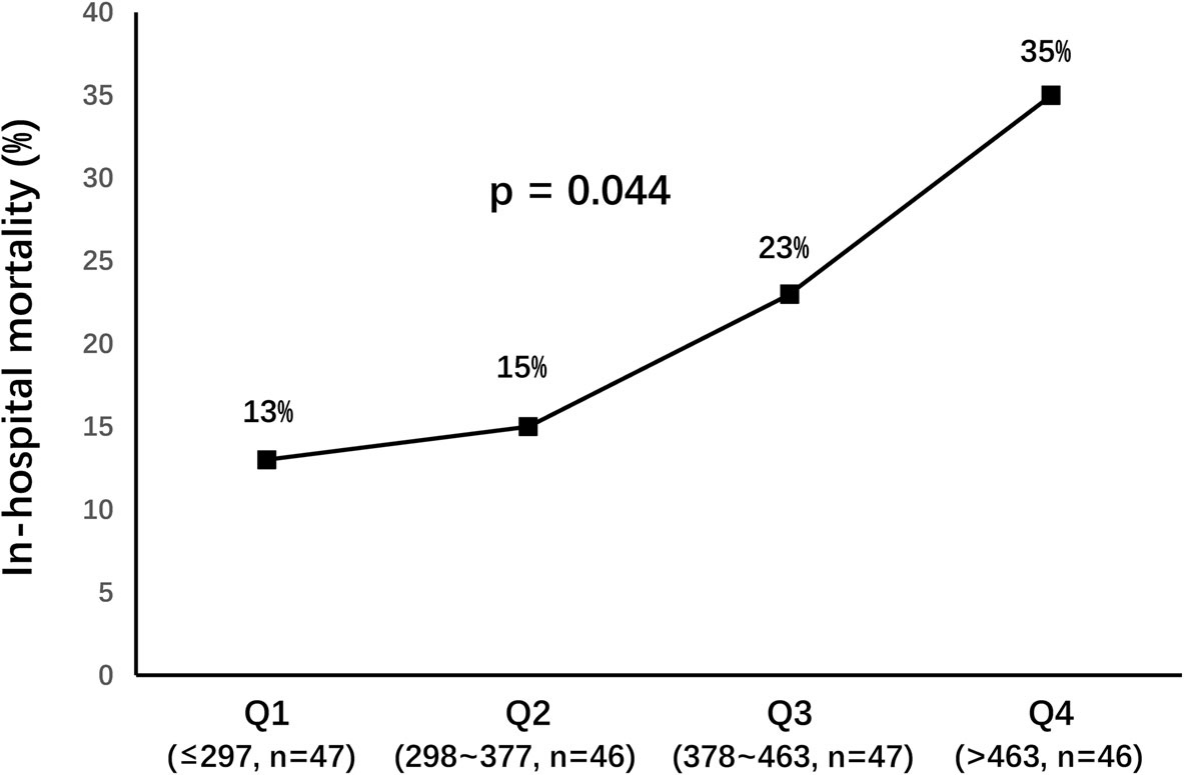
In-hospital mortality stratified by admission UA quartiles (umol/L)

In-hospital mortality was compared according to the admission serum UA quartiles. As demonstrated in Fig. 1, in-hospital mortality was significantly higher in Q4 (35%), followed by Q3 (23%), which were significantly higher than Q2 (15%) and Q1 (13%; *p* = 0.044).

### Univariate analysis and forward conditional logistic regression for in-hospital mortality

Univariate analysis indicated that there were 11 variables associated with in-hospital mortality including age, UA, D-dimer, fibrinogen, platelet count, preoperative eGFR< 60 ml/min/1.73 m^2^, extent of aortic replacement, operation time, CPB time, ACC time and HCA time with a *p* value < 0.05 (Table 2). All variables were put into forward conditional logistic regression analysis and found that age (OR = 1.063, 95% CI 1.016–1.112, *p* = 0.009), UA (OR = 1.006, 95% CI 1.002–1.010, *p* = 0.002), D-dimer (OR = 1.025, 95% CI 1.005–1.013, *p* = 0.012), operation time (OR = 1.009, 95% CI 1.005–1.013, *p* < 0.001) and extent of aortic replacement (OR = 0.412, 95% CI 0.220–0.768, *p* = 0.005) were independent risk factors of in-hospital mortality in AAAD patients (Table 3).

**Table 2:** Univariate analysis for in-hospital mortality

Clinical variables	OR (95% CI)	*p* value
Age (year)	1.040 (1.010–1.072)	**0.01**
Male	0.817 (0.350–1.910)	0.641
Hypertension	1.375 (0.670–2.820)	0.385
Diabetes	0.723 (0.082–6.372)	0.77
Prior aortic stent implant	0.442 (0.054–3.645)	0.448
Smoker	0.529 (0.245–1.139)	0.104
Onset to operation time (h)	0.988 (0.974–1.002)	0.095
Systolic blood pressure (mmHg)	0.998 (0.986–1.010)	0.699
Diastolic blood pressure (mmHg)	1.001 (0.983–1.019)	0.949
Heart rate (beat/min)	0.998 (0.980–1.017)	0.871
Ascending aorta diameter (mm)	1.004 (0.968–1.042)	0.815
Entry tears≥2	0.588 (0.126–2.741)	0.499
UA (umol/L)	1.005 (1.002–1.009)	**0.001**
Creatinine (umol/L)	1.000 (0.997–1.002)	0.965
BUN (mmol/L)	1.018 (0.939–1.103)	0.67
D-dimer (ug/mL FEU)	1.031 (1.015–1.048)	**< 0.001**
Fibrinogen (g/L)	0.653 (0.469–0.909)	**0.012**
Platelet (10^9^/L)	0.988 (0.980–0.996)	**0.004**
WBC (10^9^/L)	1.054 (0.978–1.135)	0.168
Brain ischemia	1.610 (0.397–6.531)	0.505
Coronary artery involvement	1.469 (0.641–3.365)	0.363
Lower limb ischemia	1.998 (0.933–4.276)	0.075
eGFR< 60 ml/min/1.73 m2	3.035 (1.450–6.352)	**0.003**
Extent of aortic replacement*	0.564 (0.363–0.876)	**0.011**
Bentall	0.924 (0.439–1.947)	0.836
Operation time (min)	1.010 (1.007–1.014)	**< 0.001**
CPB time (min)	1.011 (1.006–1.016)	**< 0.001**
ACC time (min)	1.007 (1.001–1.013)	**0.013**
HCA time (min)	1.034 (1.015–1.053)	**< 0.001**

The bold values indicate statistical significance

*eGFR* estimated glomerular filtration rate, *UA* uric acid, *BUN* blood urea nitrogen, *WBC* white blood cell count, *CPB* cardiopulmonary bypass, *ACC* aortic cross-clamping, *HCA* hypothermic circulatory arrest

*Categorical variable, total arch replacement was defined as “1”, hemiarch replacement was defined as “2”, ascending aortic replacement was defined as “3”

**Table 3:** Forward conditional logistic regression for in-hospital mortality

Clinical variables	OR (95% CI)	*p* value
Age (year)	1.063 (1.016–1.112)	0.009
UA (umol/L)	1.006 (1.002–1.010)	0.002
D-dimer (ug/mL FEU)	1.025 (1.005–1.013)	0.012
Operation time (min)	1.009 (1.005–1.013)	< 0.001
Extent of aortic replacement*	0.412 (0.220–0.768)	0.005

*UA* uric acid, *HCA* hypothermic circulatory arrest

*Categorical variable, total arch replacement was defined as “1”, hemiarch replacement was defined as “2”, ascending aortic replacement was defined as “3”

### ROC analysis of UA, D-dimer and age in predicting in-hospital mortality

**Fig. 2:**
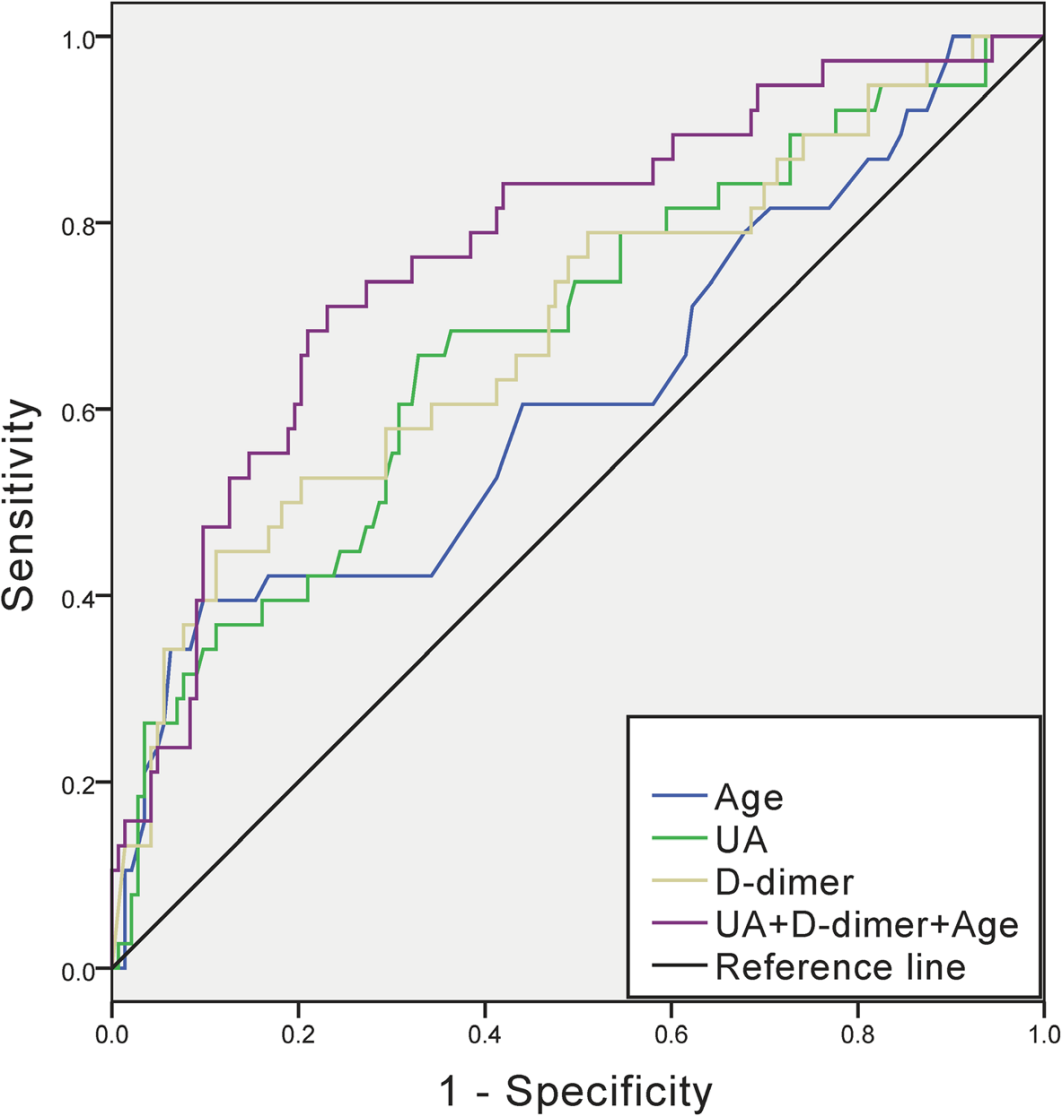
Predictive value of UA, D-dimer and Age for in-hospital mortality

ROC analysis was used to evaluate the predictive capacity of three preoperative risk factors (UA, D-dimer and age) identified by forward conditional logistic regression on in-hospital mortality, and to determine the cut-off value of these risk factors in predicting in-hospital mortality (Fig. 2). The area under the curve for UA in predicting in-hospital mortality was 0.678 (95% CI, 0.579 to 0.777; *p* = 0.001; Table 4). The best cut-off value of UA in predicting in-hospital mortality was determined to be 415 umol/L (sensitivity, 65.0%; specificity, 67.1%). The area under the curve for D-dimer in predicting in-hospital mortality was 0.689 (95% CI, 0.589 to 0.790; *p* < 0.001; Table 4). The best cut-off value of D-dimer in predicting in-hospital mortality was determined to be 24.2 μg/mL FEU (sensitivity, 44.7%; specificity, 88.8%). The area under the curve for age in predicting in-hospital mortality was 0.616 (95% CI, 0.507 to 0.724; *p* = 0.029; Table 4). The best cut-off value of age in predicting in-hospital mortality was determined to be 65 years (sensitivity, 37.5%; specificity, 90.4%). Furthermore, better predicting in-hospital death performance was shown when combining use of UA, D-dimmer and age with the area under the curve of 0.771 (Fig. 2 and Table 4).

**Table 4:** Predictive value of UA, D-dimer and Age for in-hospital mortality

	AUC	95% CI	*p* value	Cut-off value	Sensitivity (%)	Specificity (%)
UA (umol/L)	0.678	0.579–0.777	0.001	415	65.0	67.1
D-dimer (ug/mL FEU)	0.689	0.589–0.790	< 0.001	24.2	44.7	88.8
Age (years)	0.616	0.507–0.724	0.029	65	37.5	90.4
UA + D-dimer+Age	0.771	0.686–0.857	< 0.001	–	–	–

*AUC* area under the curve, *UA* uric acid

### Subgroup analysis

**Fig. 3:**
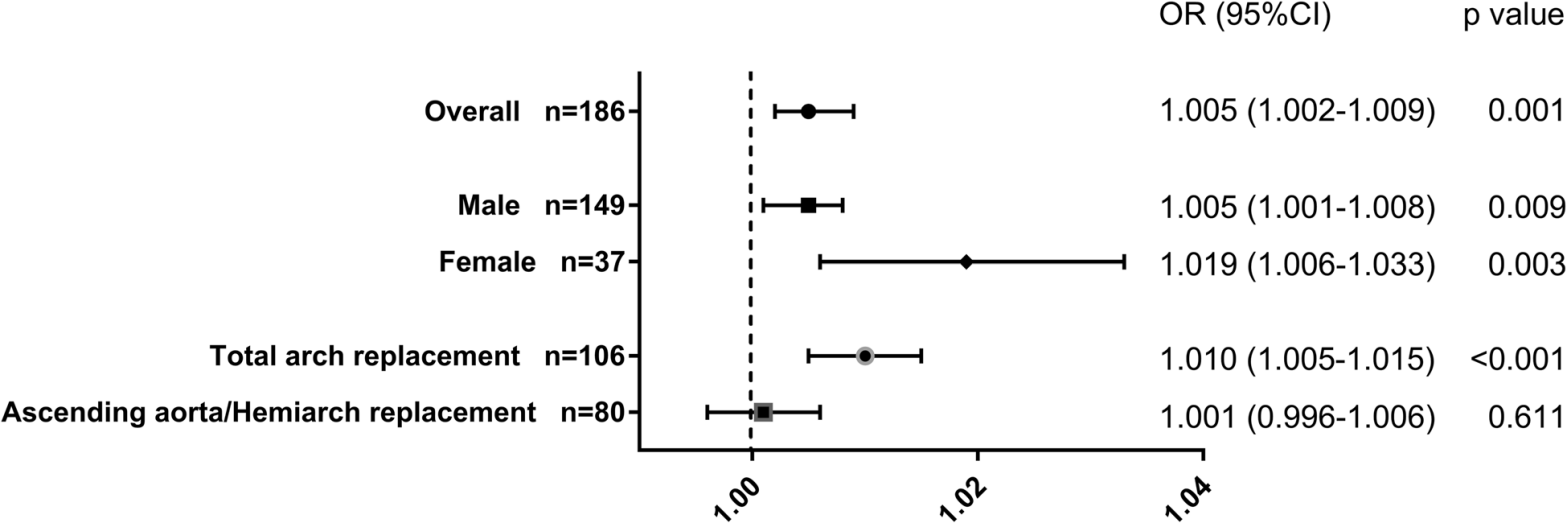
Odds ratios of UA for predicting in-hospital mortality in subgroup analysis

Subgroup analysis was performed according to sex and extent of surgery (total arch replacement or not) (Fig. 3). In the subgroup of total arch replacement, UA was significantly associated with in-hospital death (OR = 1.010, 95% CI 1.005–1.015, *p* < 0.001, power = 98.530%). While in patients underwent ascending aorta replacement or hemiarch replacement, the relationship was not significant (OR = 1.001, 95% CI 0.996–1.006, *p* = 0.611, power = 7.504%).

## Discussion

The present study was the first to evaluate the predictive value of admission serum UA on postoperative in-hospital mortality of AAAD patients. Our data indicated that admission serum UA levels were independently associated with in-hospital mortality of patients with AAAD. When UA was ≥415 umol/L, the sensitivity and specificity for in-hospital mortality was 65.0 and 67.1%, respectively. Besides, other factors including age, D-dimer, operation time and extent of aortic replacement were also found to be independently associated with in-hospital mortality, and the combination of UA, D-dimer and age could provide a stronger predictive capacity on surgical outcome.

UA is the final product of purines metabolism. Endogenous UA synthesis occurs mostly in the liver, kidney, intestine and vascular endothelium, while the exogenous purine pool derived from animal proteins and fructose catabolism [18]. Xanthine oxidoreductase is the most critical enzyme in purine metabolism, which is also one of the most important producers of reactive oxygen species (ROS) [19]. Increased level of UA is associated with increased xanthine oxidoreductase activity, which leads to increased oxidative stress [20]. UA is finally excreted by kidney and intestine.

The effects of UA on pathophysiology, morbidity and prognosis of cardiovascular disease have been indicated by a variety of studies. Several studies suggested the relationship between UA and pathological process such as oxidative stress [11], systemic inflammation [12], and activation of the renin-angiotensin system [11], which further lead to endothelial dysfunction [10], vascular smooth muscle cells proliferation [11] and increased arterial stiffness [21]. Clinical studies also indicated the association between UA and several cardiovascular diseases such as hypertension [22], coronary artery disease [23], heart failure [24] and atrial fibrillation [25]. Increased UA level was also found to be risk factor of cardiovascular disease mortality [13], poorer outcome after coronary artery bypass grafting and cardiac valve surgery [14, 15].

Several studies have suggested that serum UA levels are significantly higher among patients with aortic dissection than those in controls [17, 26]. The overall mean serum UA level in our study (386 umol/L) was consistent with that in Jiang et al. (372 umol/L). The potential mechanism underlying the correlation between UA and aortic dissection is still unclear. On the one hand, the relationship between UA level and hypertension, which is a universally accepted risk factor of aortic dissection, may explain the correlation between UA level and aortic dissection. On the other hand, it has been found that serum UA level and total antioxidant capacity were significantly associated with aortic dilatation [16], which suggested that the pro-oxidant role of UA might also participate in the pathogenesis of aortic dissection and aortic aneurysm.

The potential mechanisms underlying the predictive role of elevated serum UA on in-hospital mortality of AAAD may be multifactorial. It has been indicated that increased serum UA was an independent risk factor of renal disease [27], and it has been suggested that preoperative renal insufficiency was a stronger predictor of hospital mortality in elective aortic arch replacement surgery [28]. However, through multiple regression analysis, we found that the prognostic utility of UA was independent of renal function, which suggested that there might be independent mechanisms of UA involved in the prognosis of AAAD patients who underwent surgery. UA is converted from xanthine in an oxidation reaction catalyzed by xanthine oxidoreductase [9], and xanthine oxidoreductase is one of the most important producers of reactive oxygen species [19]. Therefore, elevated level of serum UA may identify a subgroup of patients with high xanthine oxidoreductase activity, which related to a higher state of oxidative stress, and those patients may suffer from more serious ischemia-reperfusion injury during surgical repair of AAAD. Our subgroup analysis showed that in the subgroup of total arch replacement, UA was significantly associated with in-hospital death, however, in patient underwent ascending aorta replacement or hemiarch replacement, UA was no longer associated with in-hospital death. This finding indicates a potential interaction effect of elevated UA level and deep hypothermic circulatory arrest on mortality of AAAD patients. It is widely accepted that deep hypothermic circulatory arrest causes multiple organ dysfunction through ischemia-reperfusion injury, which can increase morbidity and mortality [29]. Elevated serum UA level, which represented a higher state of oxidative stress, may identified a group of patients who are more likely to suffer from serious ischemia-reperfusion injury during deep hypothermic circulatory arrest. Further studies are needed to clarify the underlying mechanisms of UA as a biomarker for morbidity and mortality of aortic dissection. More research is required to determine whether inhibiting xanthine oxidoreductase can be an effective way to reduce mortality after surgical repair of AAAD.

The limitation of the present study is that it is a single center research with relatively small sample size. In subgroup analysis, the sample size of “ascending aorta/hemiarch replacement” group could not provide the adequate statistic power to support the negative conclusion (power = 7.504%). Thus, further multicenter studies with larger sample size were still needed to achieve a more detailed analysis.

## Conclusions

The present study shows for the first time that in patients with AAAD receiving surgical repair, elevated admission serum UA level was an independent risk factor of in-hospital mortality. Serum UA may be a promising marker for risk stratification in patients with AAAD.

## Data Availability

The datasets used and/or analyzed during the current study are available from the corresponding author on reasonable request.

## Abbreviations

AAAD: Acute type A aortic dissection
ACC: Aortic cross-clamping
AUC: Area under the receiver operating characteristic curve
CKD: Chronic kidney disease
CPB: Cardiopulmonary bypass
eGFR: Estimated glomerular filtration rate
HCA: Hypothermic circulatory arrest
ROC: Receiver operating characteristic
UA: Uric acid
WBC: White blood cell count

## References

[1] Trimarchi S, Nienaber CA, Rampoldi V (2005). Contemporary results of surgery in acute type a aortic dissection: the international registry of acute aortic dissection experience. J Thorac Cardiovasc Surg.

[2] Zierer A, El-Sayed Ahmad A, Papadopoulos N (2012). Selective antegrade cerebral perfusion and mild (28°C-30°C) systemic hypothermic circulatory arrest for aortic arch replacement: results from 1002 patients. J Thorac Cardiovasc Surg.

[3] Wen D, Du X, Dong JZ (2013). Value of D-dimer and C reactive protein in predicting inhospital death in acute aortic dissection. Heart.

[4] Huang B, Yang Y, Lu H (2015). Impact of d-dimer levels on admission on Inhospital and long-term outcome in patients with type a acute aortic dissection. Am J Cardiol.

[5] Itagaki R, Kimura N, Mieno M, et al. Characteristics and Treatment Outcomes of Acute Type A Aortic Dissection With Elevated D-Dimer Concentration. J Am Heart Assoc. 2018;7(14). 10.1161/JAHA.118.009144 PMC6064831 29987123

[6] Liu J, Sun LL, Wang J (2018). The relationship between fibrinogen and in-hospital mortality in patients with type a acute aortic dissection. Am J Emerg Med.

[7] Huang B, Tian L, Fan X (2014). Low admission platelet counts predicts increased risk of in-hospital mortality in patients with type a acute aortic dissection. Int J Cardiol.

[8] Fan X, Huang B, Lu H (2015). Impact of admission white blood cell count on short- and long-term mortality in patients with type a acute aortic dissection: an observational study. Medicine (Baltimore).

[9] Berry CE, Hare JM (2004). Xanthine oxidoreductase and cardiovascular disease: molecular mechanisms and pathophysiological implications. J Physiol.

[10] Mercuro G, Vitale C, Cerquetani E (2004). Effect of hyperuricemia upon endothelial function in patients at increased cardiovascular risk. Am J Cardiol.

[11] Corry DB, Eslami P, Yamamoto K (2008). Uric acid stimulates vascular smooth muscle cell proliferation and oxidative stress via the vascular renin-angiotensin system. J Hypertens.

[12] Ruggiero C, Cherubini A, Ble A (2006). Uric acid and inflammatory markers. Eur Heart J.

[13] Zhang W, Iso H, Murakami Y (2016). Serum uric acid and mortality form cardiovascular disease: EPOCH-JAPAN study. J Atheroscler Thromb.

[14] Hillis GS, Cuthbertson BH, Gibson PH (2009). Uric acid levels and outcome from coronary artery bypass grafting. J Thorac Cardiovasc Surg.

[15] Lazzeroni Davide, Bini Matteo, Camaiora Umberto, Castiglioni Paolo, Moderato Luca, Bosi Davide, Geroldi Simone, Ugolotti Pietro T, Brambilla Lorenzo, Brambilla Valerio, Coruzzi Paolo (2017). Serum uric acid level predicts adverse outcomes after myocardial revascularization or cardiac valve surgery. European Journal of Preventive Cardiology.

[16] Esen AM, Akcakoyun M, Esen O (2011). Uric acid as a marker of oxidative stress in dilatation of the ascending aorta. Am J Hypertens.

[17] Jiang WL, Qi X, Li X (2016). Serum uric acid is associated with aortic dissection in Chinese men. Int J Cardiol.

[18] El Ridi R, Tallima H (2017). Physiological functions and pathogenic potential of uric acid: a review. J Adv Res.

[19] Maiuolo J, Oppedisano F, Gratteri S (2016). Regulation of uric acid metabolism and excretion. Int J Cardiol.

[20] Radi R, Rubbo H, Bush K (1997). Xanthine oxidase binding to glycosaminoglycans: kinetics and superoxide dismutase interactions of immobilized xanthine oxidase-heparin complexes. Arch Biochem Biophys.

[21] Mehta T, Nuccio E, McFann K (2015). Association of Uric Acid with Vascular Stiffness in the Framingham heart study. Am J Hypertens.

[22] Cannon PJ, Stason WB, Demartini FE (1966). Hyperuricemia in primary and renal hypertension. N Engl J Med.

[23] Bos MJ, Koudstaal PJ, Hofman A (2006). Uric acid is a risk factor for myocardial infarction and stroke: the Rotterdam study. Stroke.

[24] Wannamethee SG, Papacosta O, Lennon L (2018). Serum uric acid as a potential marker for heart failure risk in men on antihypertensive treatment: the British regional heart study. Int J Cardiol.

[25] Xu X, Du N, Wang R (2015). Hyperuricemia is independently associated with increased risk of atrial fibrillation: a meta-analysis of cohort studies. Int J Cardiol.

[26] Li X, Jiang S, He J (2018). Uric acid in aortic dissection: a meta-analysis. Clin Chim Acta.

[27] Iseki K, Ikemiya Y, Inoue T (2004). Significance of hyperuricemia as a risk factor for developing ESRD in a screened cohort. Am J Kidney Dis.

[28] Okada K, Omura A, Kano H (2014). Outcome of elective total aortic arch replacement in patients with non-dialysis-dependent renal insufficiency stratified by estimated glomerular filtration rate. J Thorac Cardiovasc Surg.

[29] Allen BS, Veluz JS, Buckberg GD (2003). Deep hypothermic circulatory arrest and global reperfusion injury: avoidance by making a pump prime reperfusate--a new concept. J Thorac Cardiovasc Surg.

